# Supplementary figures and images for: Multiscale effective connectivity analysis of brain activity using neural ordinary differential equations
Source: PLoS One. 2024 Dec 4;19(12):e0314268. doi: 10.1371/journal.pone.0314268 (PMC11616886; doi:10.1371/journal.pone.0314268)

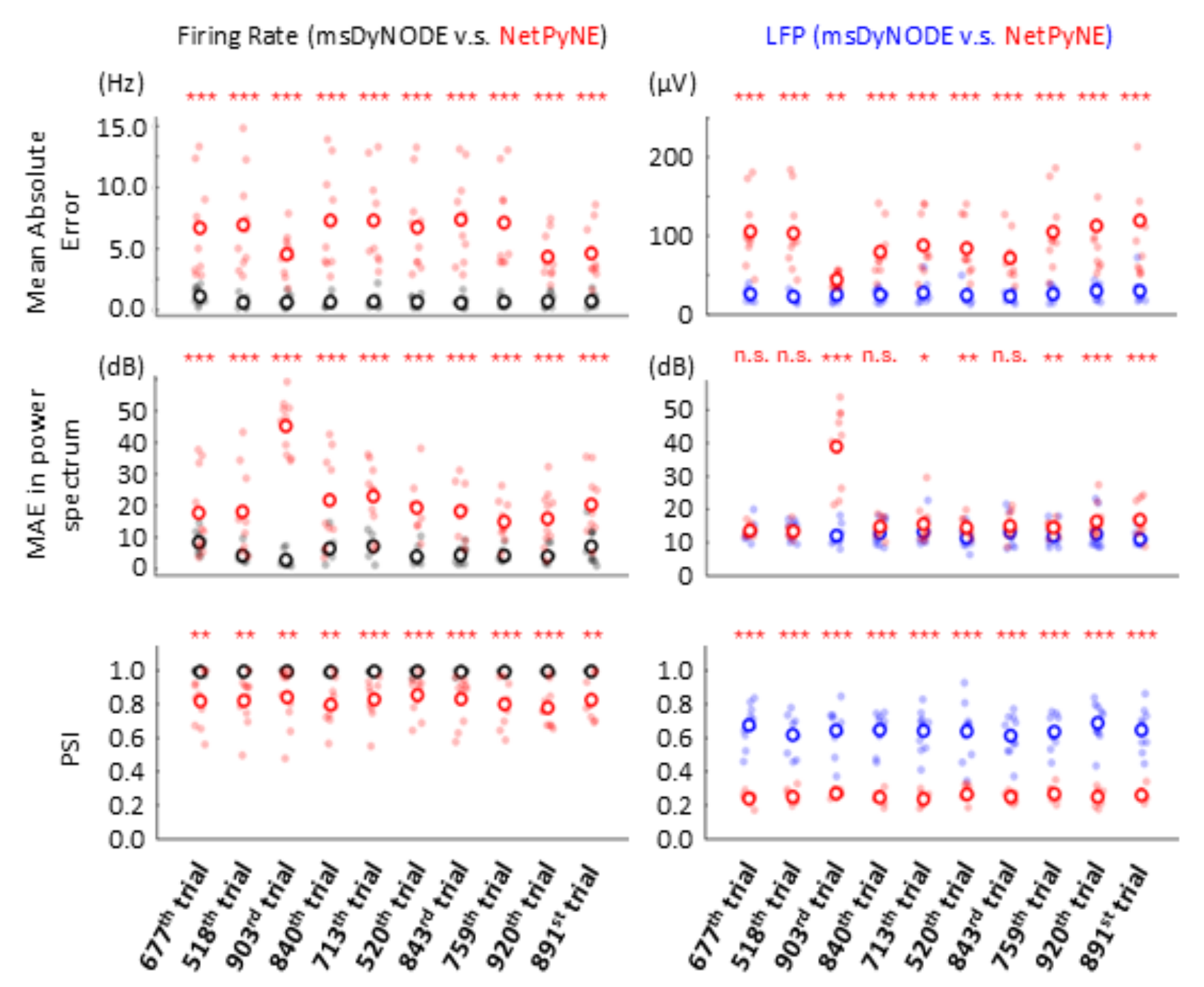

Supplement: S1 Fig — Scatter plots of MAE in the time domain, MAE in the frequency domain and PSI in the phase domain. Empty circles indicate overall average MAEs and PSI values for msDyNODE (black: firing rate, blue: LFP) and NetPyNE (red). Dim points represent average MAEs and PSI over trials for each recording channel. *p < 0.05, **p < 0.01, ***p < 0.001 using two-sided Wilcoxon’s rank-sum test. (TIF) [file pone.0314268.s001.tif]

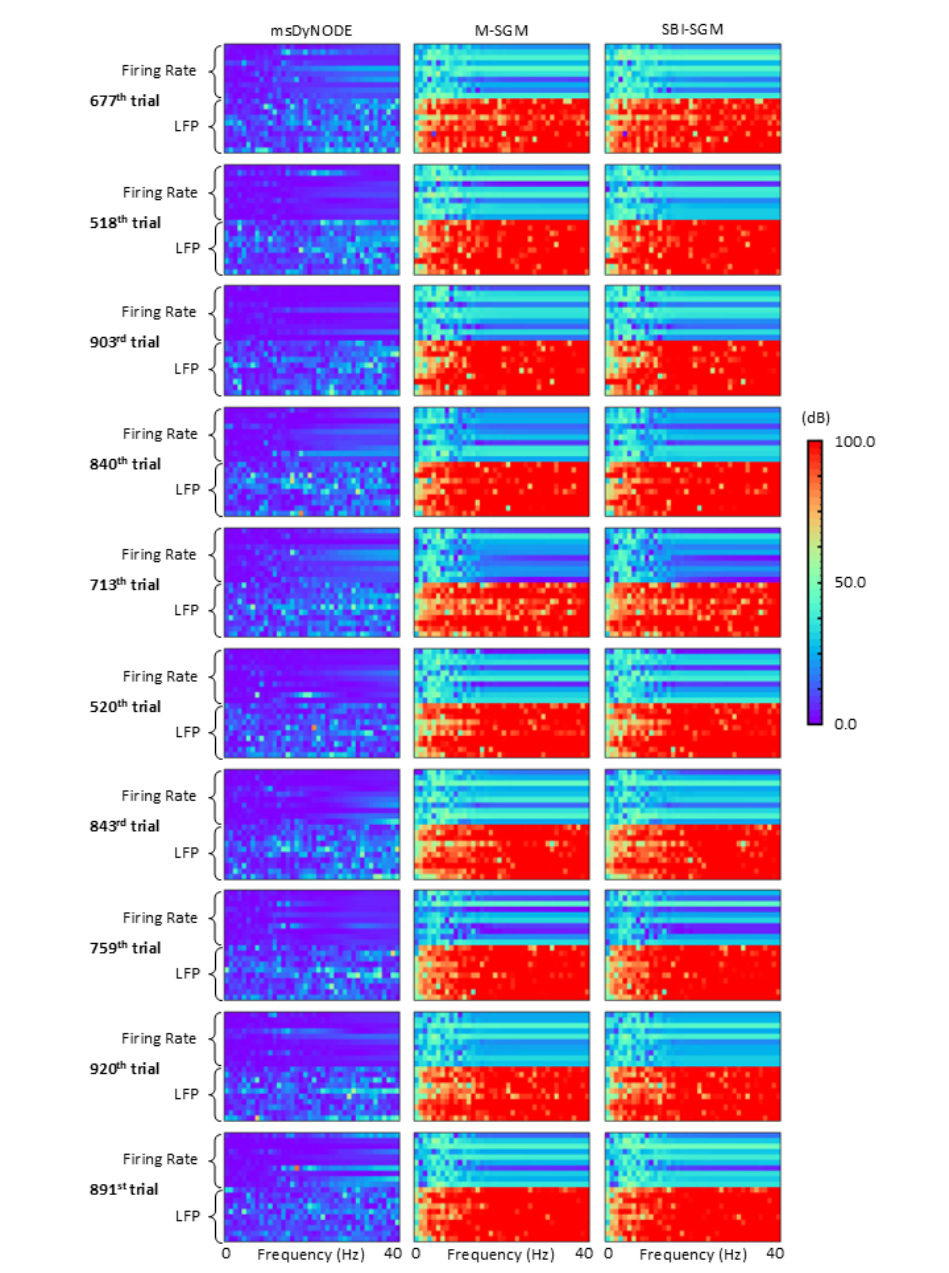

Supplement: S2 Fig — Periodograms of MAEs in frequence responses spanning from 0 to 40 Hz. (TIF) [file pone.0314268.s002.tif]

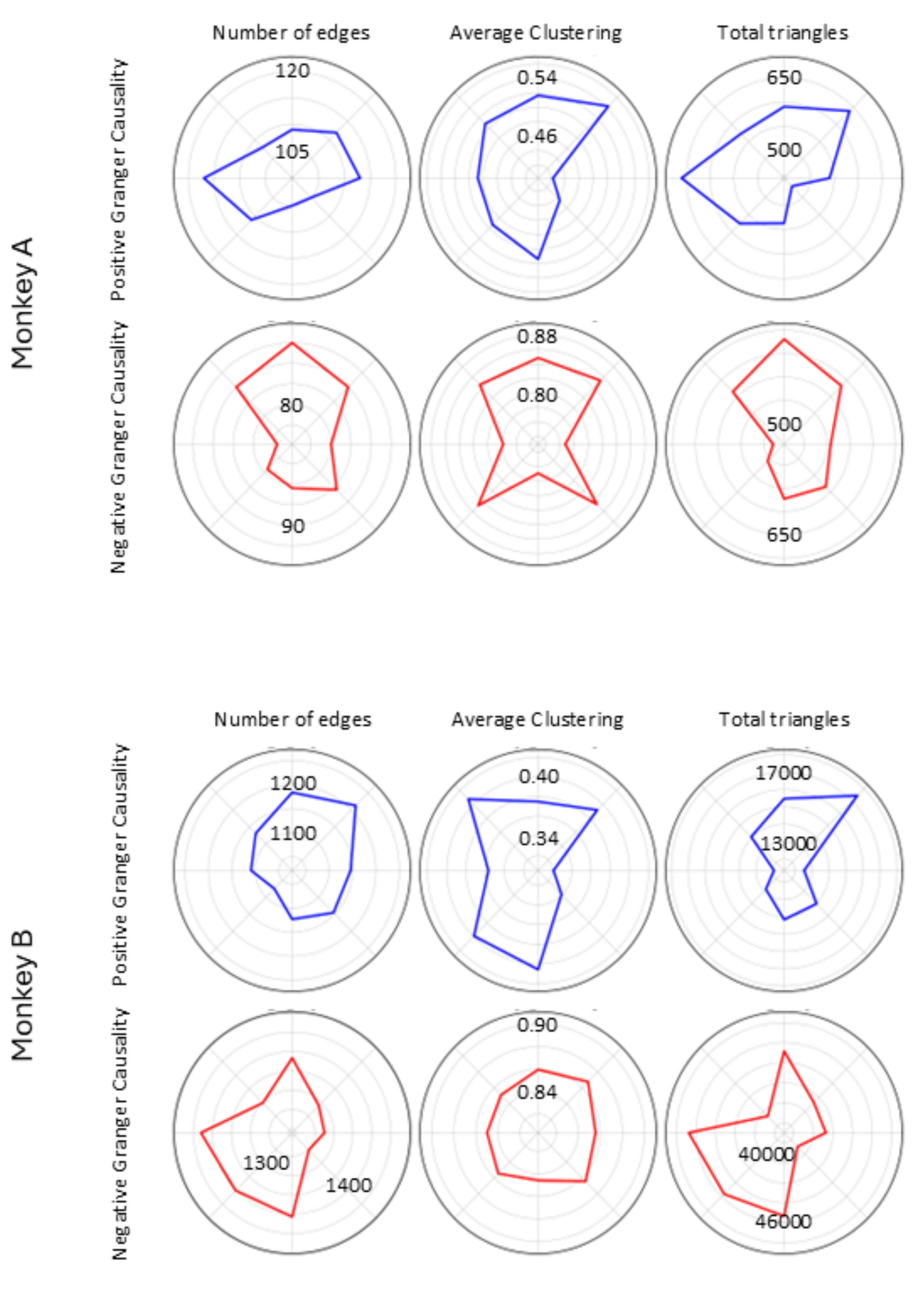

Supplement: S3 Fig — Granger causality-based graph properties over eight different target directions for Monkey A and B. Number of edges, average clustering, and number of total triangles derived from Granger causality-based excitatory (blue) and inhibitory (red) subnetworks are presented in polar coordinated for Monkey A (top) and B (bottom), respectively. (TIF) [file pone.0314268.s003.tif]

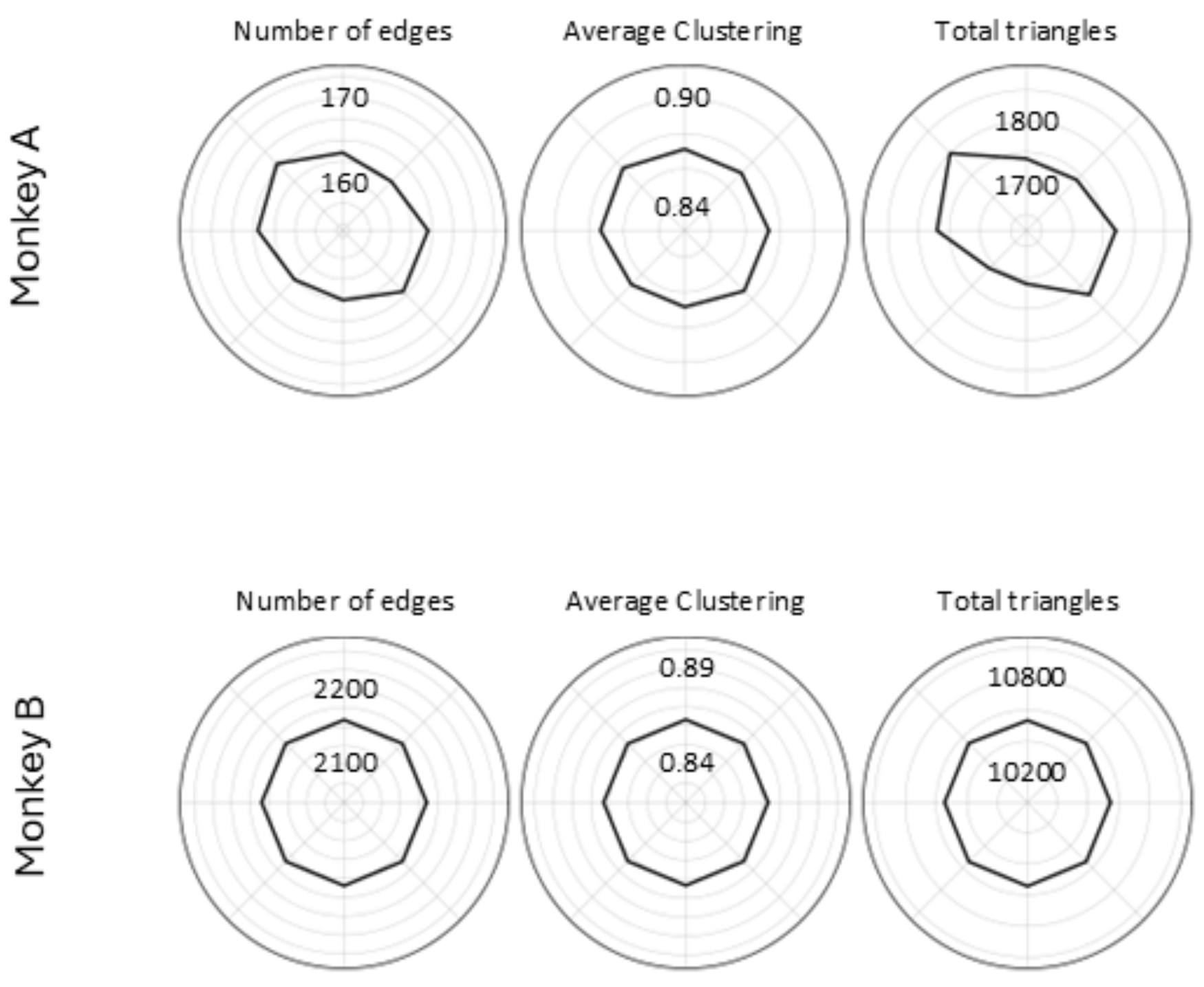

Supplement: S4 Fig — Number of edges, average clustering, and number of total triangles derived from Granger causality-based network are presented in polar coordinated for Monkey A (top) and B (bottom), respectively. (TIF) [file pone.0314268.s004.tif]
