# Supplementary material for: Multiscale effective connectivity analysis of brain activity using neural ordinary differential equations
Source: PLoS One. 2024 Dec 4;19(12):e0314268. doi: 10.1371/journal.pone.0314268 (PMC11616886; doi:10.1371/journal.pone.0314268)
Supplement: Attachment — Submitted filename: DC_101724_msDyNODE_ReviewerLetter.pdf [file pone.0314268.s005.pdf]

COCKRELL SCHOOL OF ENGINEERING
THE UNIVERSITY OF TEXAS AT AUSTIN

Department of Biomedical Engineering • 107 W. Dean Keeton St. • Austin, Texas 78712-0238

Oct 17th, 2024

RE: Manuscript ID PONE-D-24-13654 entitled "Multiscale effective connectivity analysis of brain activity using neural ordinary differential equations"

Dear reviewers,

We thank the reviewers for their valuable comments which have enabled us to develop this substantially stronger revised manuscript. The revision has been edited and reviewed in consultation with all contributing authors, and each author has approved the final form of this revision. The following is our point-by-point response to the reviewers' comments.

****

Response to Reviewer 1:

1. *This article deals with the crucial question of the multiscale nature of brain functioning. The authors proposed a tool to estimate multiscale effective connectivity via neural ordinary differential equations to model multiscale dynamics. The authors validated their model both via simulations and experimental data. The paper is well-written and easy to follow. The authors put a lot of efforts in testing their methods in different configurations (both in simulations and in real data). However, some crucial elements to demonstrate the power of their model are missing.*

Thank you for carefully reading our manuscript. We appreciate the reviewer's valuable advice that allowed us to improve our work, especially by providing more comparisons with existing methods and more metrics to demonstrate the power of our approach. Please find below our point-by-point response to your comments.

2. *First, there is no benchmark with existing estimators of causal brain connectivity (such as Granger causality, Directed Transfer Function, or Dynamic Causal Modelling [1] for instance), that could be coupled with multiplex approaches [2] to study multiscale networks.*

We thank the reviewer for this comment. In dynamical causal modeling, the brain is treated as a nonlinear dynamical system to determine the influence one neuronal system exerts over another [1]. By definition, our approach is also categorized as dynamical causal modeling. The difference between our approach and the reference indicated in the review is the method to obtain the optimal parameters, where the former utilizes deep learning techniques, and the latter uses Bayesian inference. Therefore, we posit that the dynamic causal modeling described in [1]

is not an appropriate benchmark. However, the reviewer has raised an important point regarding benchmarking our method with existing estimators causal brain connectivity. In the revised manuscript, we include new comparisons (see **Figs. S3-S4**) with the existing estimators of causal brain connectivity, including Granger causality [2] and Directed Transfer Function (DTF; [3]). Granger causality is quantified by how well a time series signal can predict another time series signal. The typical model utilized is the vector autoregression (VAR) model. Since our approach only includes one lag, the specific model implemented here for estimating Granger causality is a first-order VAR model. Then the Granger causality is determined by the log-ratio between the error variance of a reduced model and that of the full model. On the other hand, DTF is determined based on the spectral form of the multichannel autoregressive model (MVAR) with *n* channels, where we can obtain the transfer matrix of the system, ***H***. Similar to VAR in Granger causality estimation, the order of MVAR is set to be 1. Accordingly, the DTF from *i* to *j* at frequency *f*, $DTF_{ij}(f)$, is determined by the following,

$$DTF_{ij}(f) = \left|H_{ij}(f)\right| \left(\sum_{k=1}^{n} |H_{ik}(f)|^2\right)^{-1/2}$$

We apply both measures on both monkey datasets, quantify the connectivity using the common graph properties such as the number of edges, average clustering, and total triangles, and summarize them in **Figures R1-2** below. In **Figure R1**, Granger causality indicated different graph properties across the eight target directions, supporting our conclusion that both excitatory and inhibitory multiscale effective connectivity (msEC) exhibit unique patterns relating to target directions (**Figure 6c**). However, the DTF is unable to be divided into excitatory and inhibitory subnetworks. Therefore, the differences in DTF-based graph properties are relatively small across target directions (**Figure R2**), suggesting that the unique patterns in excitatory and inhibitory subnetworks cannot be observed in such an ensemble measure.

Taken together, although Granger causality and DTF are powerful tools for characterizing functional coupling between brain regions, they primarily reflect the patterns of statistical dependence. To better reveal the causal interactions that align with the actual mechanisms of brain function, it is suggested to assess effective connectivity using a mechanistic model, such as the msEC proposed in our study. We add the summarized benchmark with existing estimators of causal brain connectivity in the **Discussion** section of the revised manuscript and the **Supporting Information**.

Page 7, line 11-18 (Discussion): Although existing estimators of causal brain connectivity (e.g., Granger causality [82] and directed transfer function (DTF [83])) provide disparate graph properties (**Figs. S3-4**), Granger causality supports our observation that both excitatory and inhibitory msEC exhibit unique patterns relating to target directions. In contrast, DTF failed to demonstrate unique patterns, which may be due to its inability to be divided into excitatory and inhibitory subnetworks. While both existing estimators are powerful tools for characterizing functional coupling between brain regions, they primarily reflect the patterns of statistical dependence. To better reveal the causal interactions that align with the actual mechanisms of brain function, it is suggested to assess effective connectivity using a mechanistic model, such as msDyNODE.

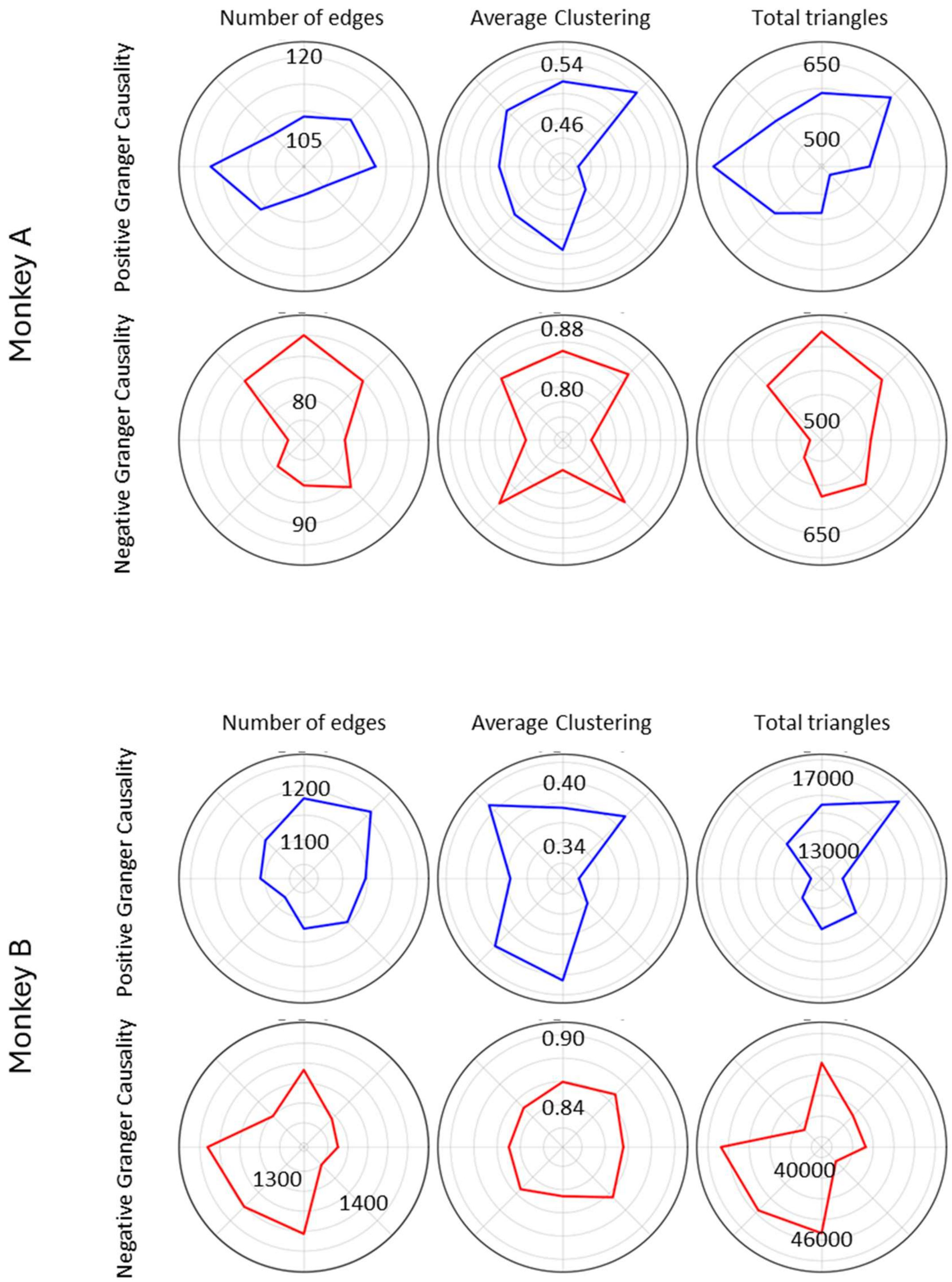

Figure R1. Granger causality-based graph properties over eight different target directions for Monkey A and B. Number of edges, average clustering, and number of total triangles derived from Granger causality-based excitatory (blue) and inhibitory (red) subnetworks are presented in polar coordinated for Monkey A (top) and B (bottom), respectively.

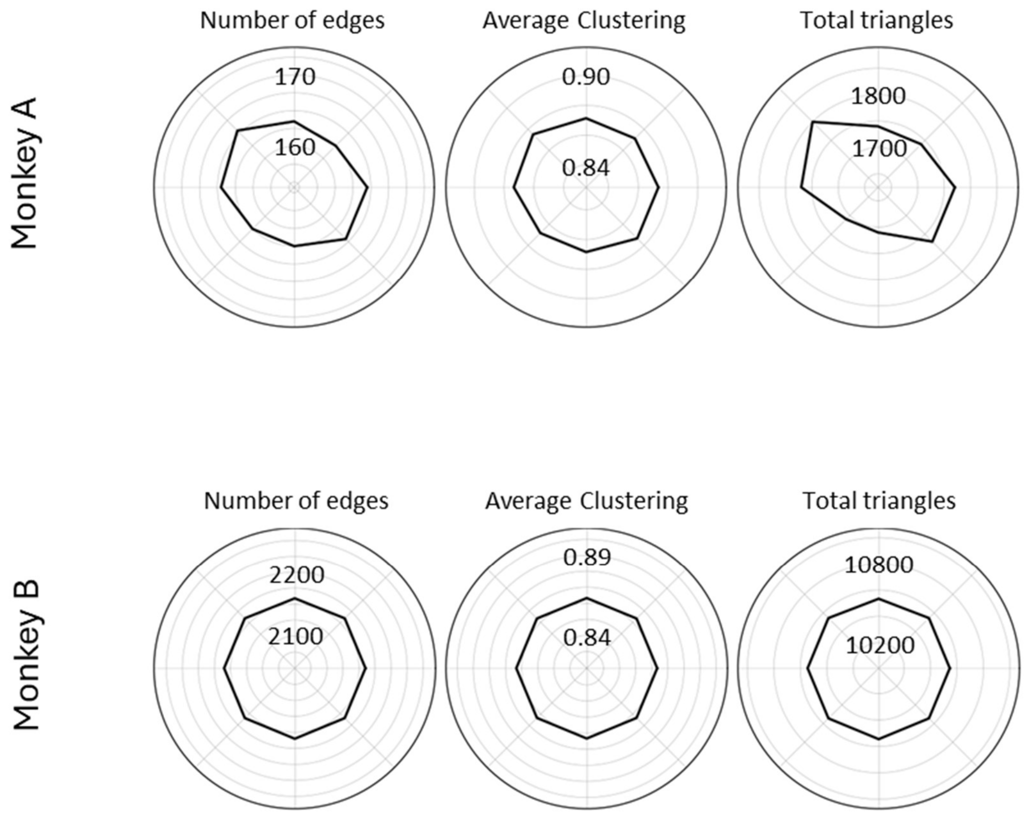

Figure R2. DTF-based graph properties over eight different target directions for Monkey A and B. Number of edges, average clustering, and number of total triangles derived from Granger causality-based network are presented in polar coordinated for Monkey A (top) and B (bottom), respectively.

3. *Secondly, there is no benchmark with existing biophysical models of brain functioning such as NetPyNE [3], or spectral graph theory-based models [4] and its Bayesian version [5]. In the latter case, the model links excitatory and inhibitory neuronal responses to the oscillatory activity that was a target identified by the authors in the manuscript.*

We thank the reviewer for raising the issue of missing benchmarks with existing biophysical models of brain functioning. To demonstrate the power of our proposed model, we apply three existing approaches: NetPyNE [4], modified spectral graph theory model (M-SGM [5]), and SGM integrated with simulation-based inference for Bayesian inference (SBI-SGM [6]), on ten randomly selected trials of Monkey A dataset and compare these model performances (**Figures R3-4**).

NetPyNE is a powerful tool to develop data-driven multiscale network models. In the network, corticostriatal neurons are considered to be excitatory populations (a total of 150 cells), and fast-spiking neurons are included for inhibitory populations (a total of 150 cells). In the simulation configuration, “LFP recording” is enabled to generate the LFP time series. To obtain the firing rate at the same LFP channel, we assign each cell to the specific LFP channel based on the distance and calculate the firing rate per channel from the spikes from the assigned cells.

We perform a grid parameter search by setting up a batch simulation in NetPyNE to find the optimal parameters that best fit the real data. As demonstrated in the revised manuscript, we compared the performance of our approach, msDyNODE, with NetPyNE in the time domain, the frequency domain, and the phase domain (**Figure R3**). The msDyNODE is superior to NetPyNE by showing smaller MAEs in both time and frequency domains, and a greater phase synchronization with the ground truths. The potential reason for relatively poor performance in NetPyNE may be due to inaccurate modeling. Indeed, NetPyNE is a powerful tool to define the model at molecular, cellular, and circuit scales when the model parameters such as populations, cell properties, and connectivity are accurate. Although NetPyNE also provides evolutionary algorithms beyond the grid parameter search to perform parameter optimization and exploration, the improper selection of parameters and their ranges to be optimized can degrade the performance.

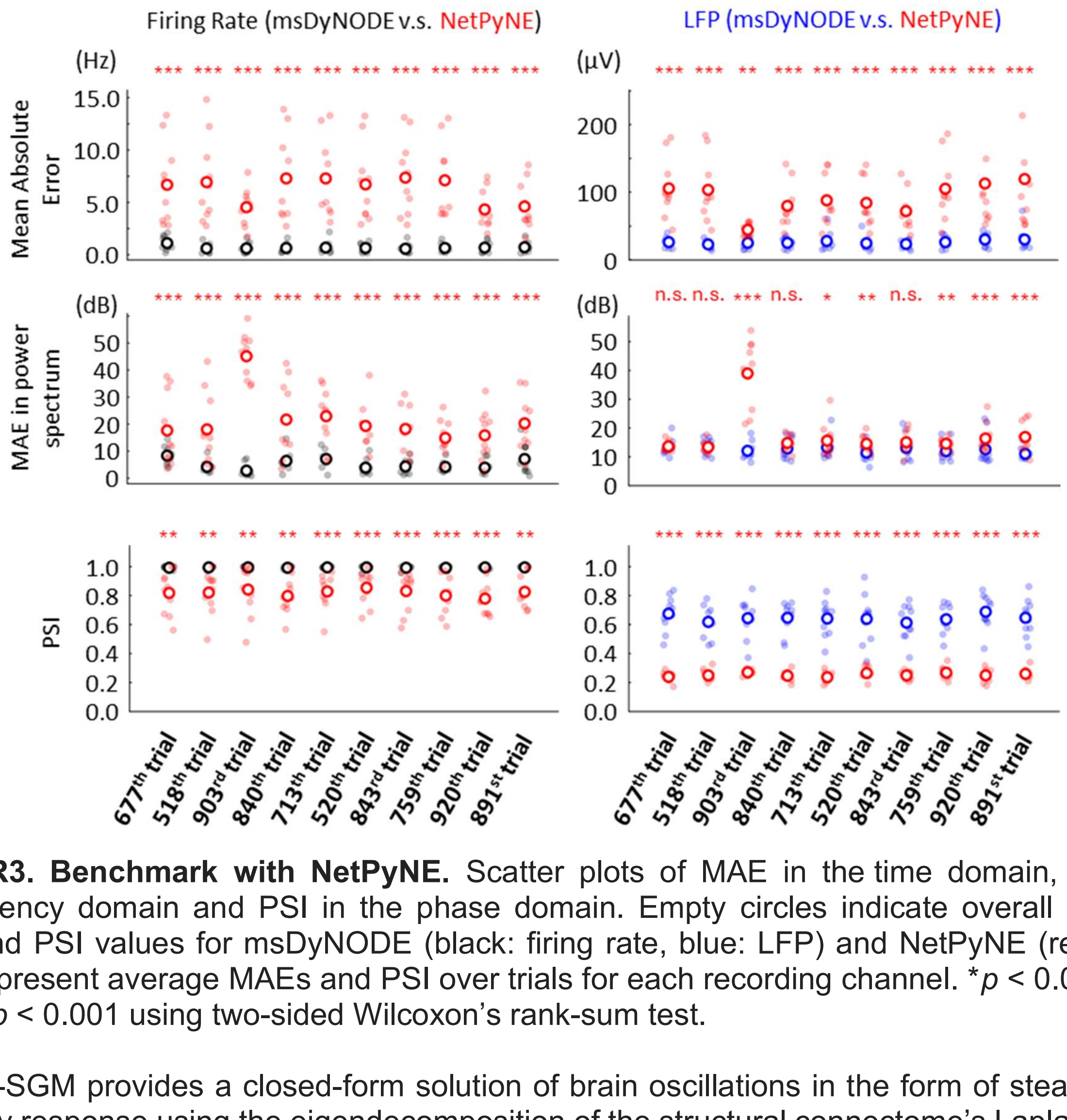

Figure R3. Benchmark with NetPyNE. Scatter plots of MAE in the time domain, MAE in the frequency domain and PSI in the phase domain. Empty circles indicate overall average MAEs and PSI values for msDyNODE (black: firing rate, blue: LFP) and NetPyNE (red). Dim points represent average MAEs and PSI over trials for each recording channel. **p* < 0.05, ***p* < 0.01, ****p* < 0.001 using two-sided Wilcoxon's rank-sum test.

M-SGM provides a closed-form solution of brain oscillations in the form of steady-state frequency response using the eigendecomposition of the structural connectome's Laplacian [7]. A dual annealing optimization [8] is performed to estimate the optimized parameters. SBI-SGM

is further proposed to infer the posterior distribution of the SGM parameters using simulation-based inference [9]. Since we do not have diffusion MRI data for structural connectivity networks, we make these parameters optimizable in the implementation. Due to the fact that SGM yields frequency responses, we compared MAE in the power spectrum with the real data for msDyNODE, M-SGM, and SBI-SGM (**Figure R4**), demonstrating that msDyNODE exhibits better performance than SGMs. msDyNODE models the real multiscale brain signals better than SGMs may be due to the consideration of nonlinear brain dynamics and spatially varying parameters.

Another advantage of msDyNODE over NetPyNE, M-SGM, and SBI-SGM is the adaptability of the new model. For msDyNODE, the user can easily modify the differential equation sections in the script. However, NetPyNE requires the development of an external module in NEURON [10,11]. For M-SGM and SBI-SGM, the new transfer function is required to derive from the new models.

In summary, the proposed approach in this study, msDyNODE, is demonstrated superior to existing biophysical models of brain functioning, including NetPyNE, M-SGM, and SBI-SGM, by showing better performance and the feasibility of customizing user needs. We add the summarized benchmark with existing biophysical models of brain functioning in the **Discussion** section of the revised manuscript and the **Supporting Information**.

Page 6, line 22-35 (Discussion): Comparing with existing biophysical models of brain functioning, including NetPyNE [61], modified spectral graph theory model (M-SGM [62]), and SGM integrated with simulation-based inference for Bayesian inference (SBI-SGM [63]), we demonstrate that msDyNODE is superior to these approaches. msDyNODE showed smaller MAEs in both time and frequency domains, and a greater phase synchronization with the ground truth signals (**Fig. S1**). The potential reason for relatively poor performance in NetPyNE may be due to inaccurate modeling. Indeed, NetPyNE is a powerful tool to define the model at molecular, cellular, and circuit scales when the model parameters such as populations, cell properties, and connectivity are accurate. Although NetPyNE also provides evolutionary algorithms beyond the grid parameter search to perform parameter optimization and exploration, the improper selection of parameters and their ranges to be optimized can degrade the performance. Furthermore, msDyNODE exhibits better performance than both versions of SGMs (**Fig. S2**). The rationale for why msDyNODE models the real multiscale brain signals better than SGMs may be due to the consideration of nonlinear brain dynamics and spatially varying parameters. Another advantage of msDyNODE over NetPyNE, M-SGM, and SBI-SGM is the adaptability of the new model. For msDyNODE, the user can easily modify the differential equation sections in the script. However, NetPyNE requires the development of an external module in NEURON [64,65]. For M-SGM and SBI-SGM, the new transfer function is required to derive from the new or customized models.

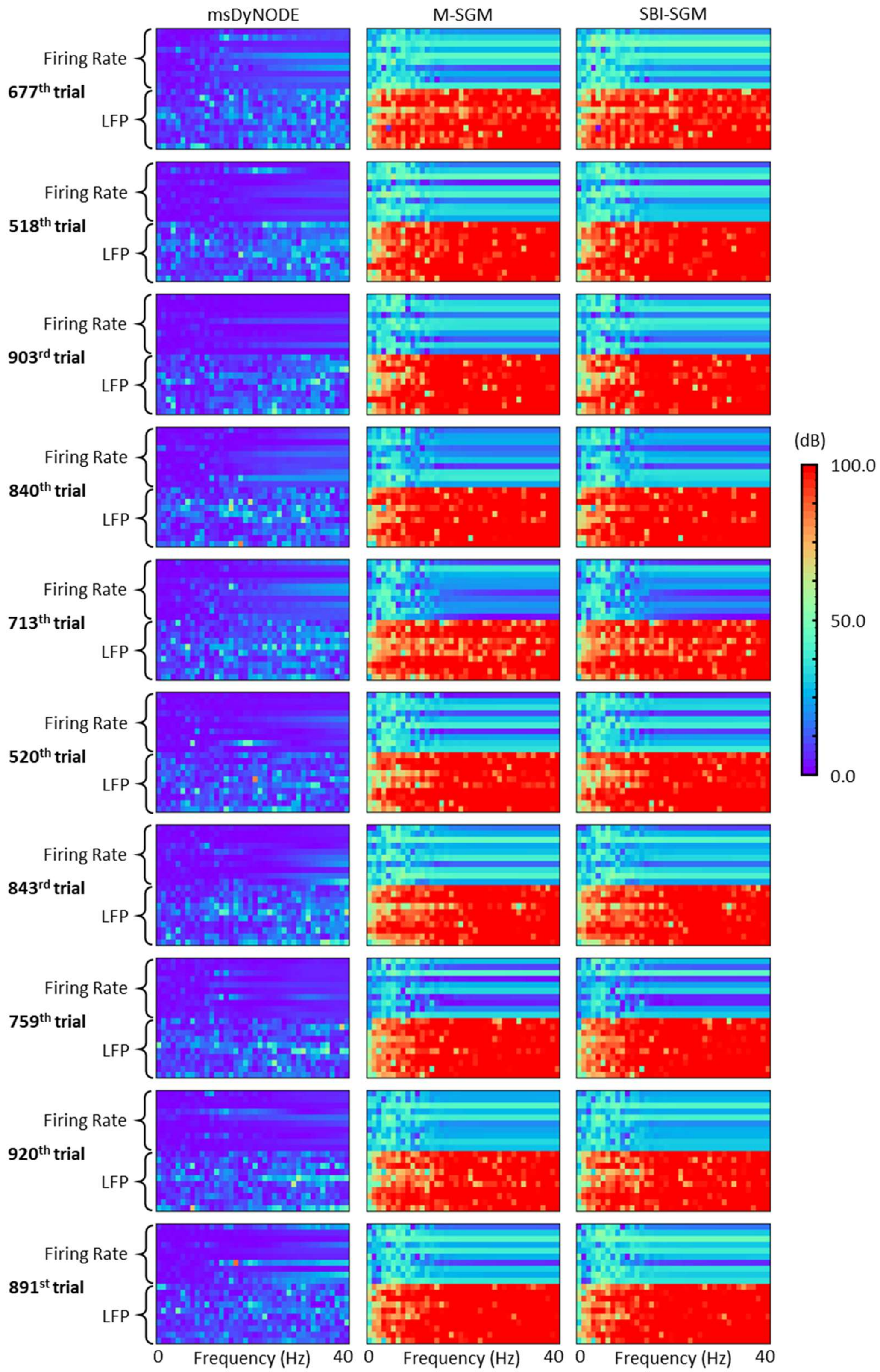

Figure R4. Benchmark with M-SGM and SBI-SGM. Periodograms of MAEs in frequence responses spanning from 0 to 40 Hz.

4. *In that spirit, additional assessments (beyond the MAE) should be proposed to better appreciate the performance of the proposed model. An evaluation of the classification performance associated to the prediction of the ground truth in the case of real data, and an assessment of the computational time to complete the analysis.*

We appreciate the reviewer's point on providing additional assessment beyond MAE. Given good performances in the time domain based on MAE, it is expected that the proposed model maintains the integrity of information represented by the neural activity, and thus the classifier trained with the real data and that trained with the model prediction can yield the comparable decoding capability. Evaluation of the classification accuracy on real data and model predictions may not add value to the performance of the proposed model. Instead, we provide two additional assessments on different domains: frequency and phase. In frequency domain, we assess the model performance in the frequency domain by calculating the MAE of the power spectrum. In phase domain, we evaluate whether the predicted and the real signals are phase-synchronized by calculating the phase synchrony index (PSI [12]). We first utilized the Hilbert transform, **HT**[·], to obtain the instantaneous phase of two time series signals, $\mathbf{\Phi}_1(t)$ and $\mathbf{\Phi}_2(t)$, from the pair of signals, $\mathbf{s}_1(t)$ and $\mathbf{s}_2(t)$.

$$z_i(t) = s_i(t) + j\,\mathbf{HT}[s_i(t)] = \mathbf{A}_i(t)e^{j\mathbf{\Phi}_i(t)}$$

$$\mathbf{HT}[s_i(t_k)] = s_i(t_k) * \frac{1}{2\pi}\left[\int_{-\pi}^{0} j \cdot e^{jwk}dw - \int_{0}^{\pi} j \cdot e^{jwk}dw\right]$$

where k = 1 to T, $\mathbf{A}_i(t)$ represents the instantaneous amplitude, and $\mathbf{\Phi}_i(t)$ represents the instantaneous phase. The instantaneous phase synchronous (IPS), which measured the phase similarity at each timepoint, can be calculated by the following,

$$IPS(t) = 1 - \sin\left(\frac{|\mathbf{\Phi}_1(t) - \mathbf{\Phi}_2(t)|}{2}\right)$$

where the phase is in the unit of degree. IPS spans the range of 0-1, where a larger value indicates a stronger synchrony. We then define a quarter of the whole range of phase difference (180°), 45°, as the threshold. When the phase difference is less than 45°, IPS was greater than 0.62, thus revealing a better performance. We finally calculated the PSI by the ratio of the time with the IPS greater than 0.62,

$$PSI = \frac{t_{IPS} > 0.62}{T}$$

These two measures are both applied to both monkey datasets, and the results are summarized in **Figure R5**. Overall, the msDyNODE well captures the signal's power across 0 to 40 Hz for both Monkey A (**Figure R5a-b**) and Monkey B (**Figure R5d-e**). In addition, msDyNODE-predictions are in sync with the ground truths by showing most of the predictions with PSI greater than 0.5 (**Figure R5c,f**). The model performance in the frequency and phase domains align well with that in the time domain, where the msDyNODE predicts LFP better with more channels included in the model.

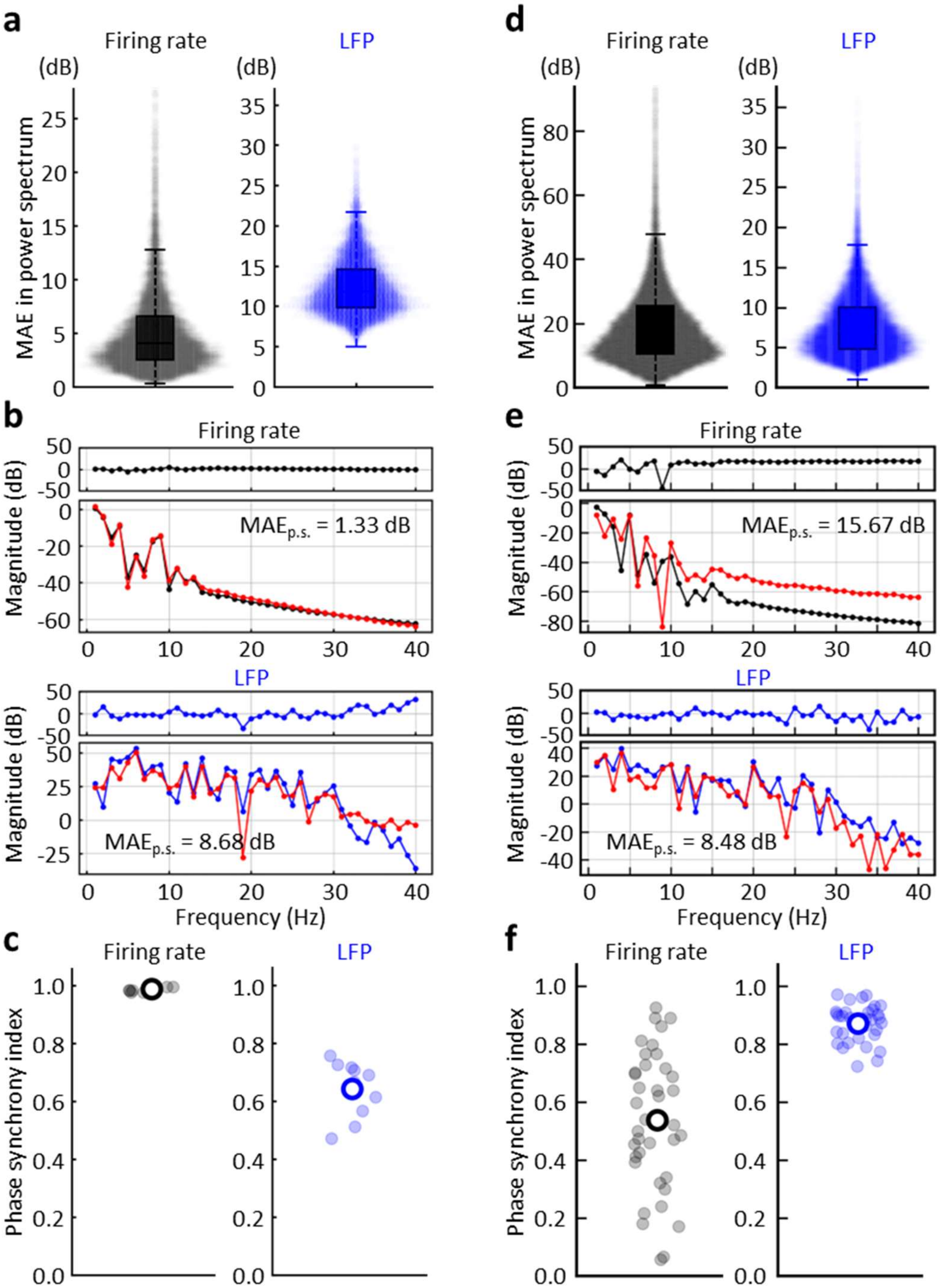

Figure R5. Additional model assessments in the frequency and phase domains for Monkey A (a-c) and Monkey B (d-f). (**a**) Boxplots and swarmplots of the mean absolute errors in power spectrum for firing rates and LFPs. (**b**) The representative power spectrum from GT and msDyNODE with the selected example in **Fig. 4b**. (**c**) Scatter plots of PSI values for firing rates and LFPs. Empty circles indicate overall average PSI values. Dim points represent average PSI over trials for each recording channel. (**d-f**) The same in (**a-c**) but for disparate dataset.

Despite accurate modeling of multiscale neural activity, the major limitation of the proposed approach is the network training, which is computationally expensive and has long training times (on the order of hours). In contrast, the prediction process is significantly faster, which takes several minutes to complete the prediction. To mitigate the issue on long network training time, transfer learning [13] from a previously trained network can potentially speed up the convergence of the learning process. In the future work, we will investigate how transfer learning techniques can improve the computation time to complete the analysis.

In summary, we have included additional assessments on the performance of the model, including the predictions in the frequency and phase domains and the computational time, in the **Results**, **Discussion**, and **Materials and Methods** sections. **Figure R5** is integrated into original **Figs. 4 and 5** in the revised manuscript.

Page 4, line 20-25 (Results): Beyond MAE in the time domain, we also assess MAE in the frequency domain and phase synchronization in the phase domain (**Fig. 4f-h, Fig. 5f-h**; see **Materials and Methods**). Overall, the msDyNODE captures the signal's power for both Monkey A (**Fig. 4f,g**) and Monkey B (**Fig. 5f,g**). Notably, phase synchronization is recognized as a fundamental neural mechanism that supports neural communication and plasticity [40]. Therefore, the model performance in the phase domain is crucial. We demonstrated that msDyNODE-predictions are in sync with ground truth by showing most of the predictions have a phase synchrony index greater than 0.5 (**Fig. 4h, Fig. 5h**).

Page 7, line 3-6 (Discussion): Furthermore, hours of network training time are another issue for quick implementation. In future work, transfer learning [76] from the previously trained network may be a possible strategy to improve computation time by potentially speeding up the convergence of the learning process.

5. *Finally, further information on the deep learning architecture that has been chosen would be important to understand all the steps that are required to build the model.*

We thank the reviewer for this comment on further clarification of the deep learning architecture that has been chosen for msDyNODE. Deep learning is a powerful technique that can automatically learn complex tasks with large amounts of data, and it is tailored for specific tasks by implementing different architectures. Common examples are convolutional neural networks for image recognition [14], recurrent neural networks for sequential data [15], transformers for natural language processing tasks [16], and generative adversarial networks for generating authentic new data [17] and denoising [18]. RNN and its variants (such as regularized RNN, long short-term memory [19], and gated recurrent unit [20]) seem to be good deep-learning techniques for implementation. However, as mentioned in the Discussion section, it may fail to capture faster dynamics information and can introduce artifacts when matching the sampling rates between signals. Unlike RNN, neural ordinary differential equations (NODE [21]) chosen in this study naturally model the continuously-defined dynamics, indicating that both slow and fast dynamics information can be captured. This capability is crucial for multiscale modeling since the system dynamics the researchers focus on vary at different scales. Additionally, NODE allows us to define the multiscale system by customizing the differential equations in the network. After the model training, we are able to investigate the physiological interpretation of the multiscale system by extracting the model parameters. Here we add additional comments on

the selection of the deep learning architecture in the **Discussion** section of the revised manuscript.

Page 6, line 3-6 (Discussion): Different deep learning architectures are tailored for specific tasks. Common examples include convolution neural networks for image recognition [52], recurrent neural networks (RNN) for sequential data [53], transformers for natural language processing tasks [54], and generative adversarial networks for generating authentic new data [55] and denoising [56].

Page 6, line 10-13 (Discussion): …, indicating that both slow and fast dynamics can be captured. Such a capability is crucial for multiscale modeling since the system dynamics vary at different scales. Additionally, NODE allows us to define the multiscale system by customizing the differential equations in the network, in which we can investigate the physiological interpretation of the modeled systems.

6. *Could the author update the GitHub repository please? With the current version of the files, it is not possible to load the data for the toy example.*

We thank the reviewer for this suggestion. We are currently updating the GitHub repository so that it provides a toy example for the public to access and customize their implementations before the manuscript is officially published.

References

1. Friston KJ, Harrison L, Penny W. Dynamic causal modelling. NeuroImage. 2003 Aug 1;19(4):1273–302.

2. Granger CWJ. Investigating Causal Relations by Econometric Models and Cross-spectral Methods. Econometrica. 1969;37(3):424–38.

3. Kaminski MJ, Blinowska KJ. A new method of the description of the information flow in the brain structures. Biol Cybern. 1991 Jul 1;65(3):203–10.

4. Dura-Bernal S, Suter BA, Gleeson P, Cantarelli M, Quintana A, Rodriguez F, et al. NetPyNE, a tool for data-driven multiscale modeling of brain circuits. Bhalla US, Calabrese RL, Sterratt D, Wójcik DK, editors. eLife. 2019 Apr 26;8:e44494.

5. Verma P, Nagarajan S, Raj A. Spectral graph theory of brain oscillations—-Revisited and improved. NeuroImage. 2022 Apr 1;249:118919.

6. Jin H, Verma P, Jiang F, Nagarajan SS, Raj A. Bayesian inference of a spectral graph model for brain oscillations. NeuroImage. 2023 Oct 1;279:120278.

7. Raj A, Cai C, Xie X, Palacios E, Owen J, Mukherjee P, et al. Spectral graph theory of brain oscillations. Human Brain Mapping. 2020;41(11):2980–98.

8. Xiang Y, Sun DY, Fan W, Gong XG. Generalized simulated annealing algorithm and its application to the Thomson model. Physics Letters A. 1997 Aug 25;233(3):216–20.

9. Cranmer K, Brehmer J, Louppe G. The frontier of simulation-based inference. Proceedings of the National Academy of Sciences. 2020 Dec;117(48):30055–62.

10. Carnevale NT, Hines ML. The NEURON book. Cambridge University Press; 2006.

11. Tikidji-Hamburyan RA, Narayana V, Bozkus Z, El-Ghazawi TA. Software for Brain Network Simulations: A Comparative Study. Front Neuroinform [Internet]. 2017 Jul 20 [cited 2024 Oct 17];11. Available from: https://www.frontiersin.org/journals/neuroinformatics/articles/10.3389/fninf.2017.00046/full

12. Chang YJ, Chen YI, Yeh HC, Santacruz SR. Neurobiologically realistic neural network enables cross-scale modeling of neural dynamics. Sci Rep. 2024 Mar 1;14(1):5145.

13. Pan SJ, Yang Q. A survey on transfer learning. IEEE Transactions on knowledge and data engineering. 2010;22(10):1345–59.

14. Chauhan R, Ghanshala KK, Joshi RC. Convolutional Neural Network (CNN) for Image Detection and Recognition. In: 2018 First International Conference on Secure Cyber Computing and Communication (ICSCCC) [Internet]. 2018 [cited 2024 Oct 17]. p. 278–82. Available from: https://ieeexplore.ieee.org/abstract/document/8703316

15. Pan Y, Wang J. Model Predictive Control of Unknown Nonlinear Dynamical Systems Based on Recurrent Neural Networks. IEEE Transactions on Industrial Electronics. 2012 Aug;59(8):3089–101.

16. Wolf T, Debut L, Sanh V, Chaumond J, Delangue C, Moi A, et al. Transformers: State-of-the-Art Natural Language Processing. In: Liu Q, Schlangen D, editors. Proceedings of the 2020 Conference on Empirical Methods in Natural Language Processing: System Demonstrations [Internet]. Online: Association for Computational Linguistics; 2020 [cited 2024 Oct 17]. p. 38–45. Available from: https://aclanthology.org/2020.emnlp-demos.6

17. Goodfellow I, Pouget-Abadie J, Mirza M, Xu B, Warde-Farley D, Ozair S, et al. Generative Adversarial Nets. In: Advances in Neural Information Processing Systems [Internet]. Curran Associates, Inc.; 2014 [cited 2023 Apr 26]. Available from: https://papers.nips.cc/paper_files/paper/2014/hash/5ca3e9b122f61f8f06494c97b1afccf3-Abstract.html

18. Chen YI, Chang YJ, Liao SC, Nguyen TD, Yang J, Kuo YA, et al. Generative adversarial network enables rapid and robust fluorescence lifetime image analysis in live cells. Commun Biol. 2022 Jan 11;5(1):1–11.

19. Hochreiter S, Schmidhuber J. Long Short-Term Memory. Neural Computation. 1997 Nov 1;9(8):1735–80.

20. Chung J, Gulcehre C, Cho K, Bengio Y. Empirical Evaluation of Gated Recurrent Neural Networks on Sequence Modeling [Internet]. arXiv; 2014 [cited 2024 Oct 17]. Available from: http://arxiv.org/abs/1412.3555

21. Chen RTQ, Rubanova Y, Bettencourt J, Duvenaud D. Neural Ordinary Differential Equations. arXiv:180607366 [cs, stat] [Internet]. 2019 Dec 13 [cited 2022 Apr 28]; Available from: http://arxiv.org/abs/1806.07366
